# Association of dietary magnesium intake and magnesium depletion score with psoriasis among US adults: A cross-sectional NHANES study

**DOI:** 10.7555/JBR.39.20250386

**Published:** 2026-09-28

**Authors:** Qinyang Cheng, Jiliang Lu, Chaojing Zhou, Chenxingyue Zhang, Ziyue Diao, Zhiqiang Yin

**Affiliations:** Department of Dermatology, the First Affiliated Hospital of Nanjing Medical University, Nanjing, Jiangsu 210029, China

Dear Editor,

Psoriasis is a chronic systemic inflammatory disorder that clinically manifests as erythematous plaques covered with silvery scales^[1–2]^. Magnesium is an essential mineral nutrient, serving as a crucial cofactor for hundreds of enzymatic reactions. It is fundamental to energy metabolism, protein and DNA synthesis, nerve transmission, muscle function, and blood glucose regulation^[3]^. Low levels of magnesium have been associated with numerous chronic diseases, including Alzheimer's disease, insulin resistance, and type 2 diabetes mellitus^[4]^. However, the epidemiological link between magnesium and psoriasis remains unclear, partly because serum magnesium levels do not accurately reflect cellular deficiency. To circumvent the limitations of serum measurements, Fan and colleagues developed the Magnesium Depletion Score (MDS)^[5]^, a composite score that assesses the risk of magnesium deficiency by evaluating factors that impair kidney reabsorption. The current study investigates the association between MDS, dietary magnesium intake, and odds of psoriasis, an aspect that has not been previously explored.

Data from the National Health and Nutrition Examination Survey (NHANES) cycles 2003–2006 and 2009–2014 were analyzed (https://www.cdc.gov/nchs/nhanes/). The MDS consists of: (1) current diuretic use (1 point); (2) current proton pump inhibitor use (1 point); (3) renal function (1 point for estimated glomerular filtration rate [eGFR] 60–< 90 mL/[min·1.73 m^2^] and 2 points for eGFR < 60 mL/[min·1.73 m^2^]); and (4) heavy alcohol consumption (1 point), defined as more than one drink per day for females or more than two drinks per day for males^[5]^. In the current study, MDS values were categorized into four groups: Q1 (MDS = 0), Q2 (MDS = 1), Q3 (MDS = 2), and Q4 (MDS ≥ 3), consistent with previous methods^[6]^. Dietary magnesium intake was calculated as the mean of two 24-hour dietary recall interviews. Covariates included sociodemographic and health behavioral characteristics. For normally distributed continuous variables, data were presented as mean ± standard deviation (SD) and analyzed using Student's *t*-tests; for categorical variables, data were expressed as frequencies and percentages and analyzed using chi-square tests. Associations between dietary magnesium intake, MDS, and psoriasis were evaluated using multivariate logistic regression. Restricted cubic splines (RCS) were employed to investigate potential nonlinear relationships between dietary magnesium intake, MDS, and psoriasis. Subgroup analyses were conducted to identify factors influencing the association between MDS and psoriasis. All analyses incorporated the appropriate NHANES sampling weights, strata, and primary sampling units to account for the complex survey design. All analyses were performed using R (version 4.4.1), with *P* < 0.05 considered statistically significant.

The analysis included 17823 participants—511 cases of psoriasis and 17312 controls—drawn from the NHANES data for the years 2003–2006 and 2009–2014. Psoriasis cases showed a significantly higher prevalence in the highest MDS category compared with controls (Q4: 8.00% *vs.* 4.81%; *P* < 0.001), whereas dietary magnesium intake did not differ significantly between the two groups (***Table 1***). When the MDS was categorized into four groups, with Q1 serving as the reference, the Q4 MDS group was associated with significantly higher odds of psoriasis in the fully adjusted model (odds ratio [OR] = 1.97; 95% confidence interval [CI], 1.14–3.45; *P* = 0.046; ***Supplementary Table 1***). The RCS analysis suggested a nonlinear association between MDS and the odds of psoriasis (*P* for nonlinearity = 0.007; ***Fig. 1***), with the association becoming more pronounced at higher MDS values. In subgroup analyses, the association between higher MDS and psoriasis was statistically significant among individuals aged 20–40 years, females, and participants with a normal BMI (***Fig. 2***). However, evidence of effect modification was observed only for race/ethnicity (*P* for interaction = 0.02).

**Table 1:** Demographics and characteristics of participants from NHANES 2003–2006 and 2009–2014

Characteristics^a^	Total (*n* = 17823)	Non-psoriasis (*n* = 17312)	Psoriasis (*n* = 511)	*P*
Age (years)				0.096
[20, 40)	6693 (40.10%)	6534 (40.30%)	159 (33.96%)	
[40, 60)	6867 (42.25%)	6655 (42.20%)	212 (43.90%)	
≥ 60	4263 (17.65%)	4123 (17.50%)	140 (22.13%)	
Sex				0.203
Female	9102 (50.89%)	8833 (51.01%)	269 (47.47%)	
Male	8721 (49.11%)	8479 (48.99%)	242 (52.53%)	
Race				< 0.001
White	8257 (68.71%)	7942 (68.32%)	315 (80.46%)	
Black	3674 (11.13%)	3607 (11.30%)	67 (6.01%)	
Other race	5892 (20.16%)	5763 (20.38%)	129 (13.53%)	
Education				0.262
Under high school	1556 (4.53%)	1528 (4.58%)	28 (3.02%)	
High school	2501 (10.59%)	2436 (10.63%)	65 (9.34%)	
Over high school	13766 (84.88%)	13348 (84.79%)	418 (87.65%)	
BMI (kg/m^2^)				0.193
Normal	5270 (31.74%)	5152 (31.92%)	118 (26.20%)	
Overweight	5862 (32.90%)	5692 (32.82%)	170 (35.43%)	
Obese	6691 (35.36%)	6468 (35.26%)	223 (38.37%)	
Smoking				< 0.001
Never	9866 (54.99%)	9641 (55.43%)	225 (41.74%)	
Former	4063 (23.17%)	3893 (22.71%)	170 (37.01%)	
Current	3894 (21.85%)	3778 (21.87%)	116 (21.25%)	
Alcohol				0.287
No	15675 (84.97%)	15233 (85.04%)	442 (82.61%)	
Yes	2148 (15.03%)	2079 (14.96%)	69 (17.39%)	
Diabetes				0.149
No	15041 (88.86%)	14630 (88.95%)	411 (86.04%)	
Yes	2782 (11.14%)	2682 (11.05%)	100 (13.96%)	
Dietary magnesium (mg)	282.50 ± 135.22	282.00 ± 135.72	297.00 ± 129.10	0.052
MDS category				< 0.001
Q1	8566 (46.21%)	8374 (46.65%)	192 (32.99%)	
Q2	5820 (35.63%)	5628 (35.34%)	192 (44.47%)	
Q3	2373 (13.24%)	2290 (13.19%)	83 (14.54%)	
Q4	1064 (4.92%)	1020 (4.81%)	44 (8.00%)	
^**a**^Continuous variables are reported as mean ± standard deviation, and categorical variables are expressed as unweighted *n* (weighted %). *P* < 0.05 is considered statistically significant. For MDS category, Q1 indicates MDS = 0, Q2 indicates MDS = 1, Q3 indicates MDS = 2, and Q4 indicates MDS ≥ 3. Abbreviations: BMI, body mass index; MDS, magnesium depletion score.				

**Figure 1:**
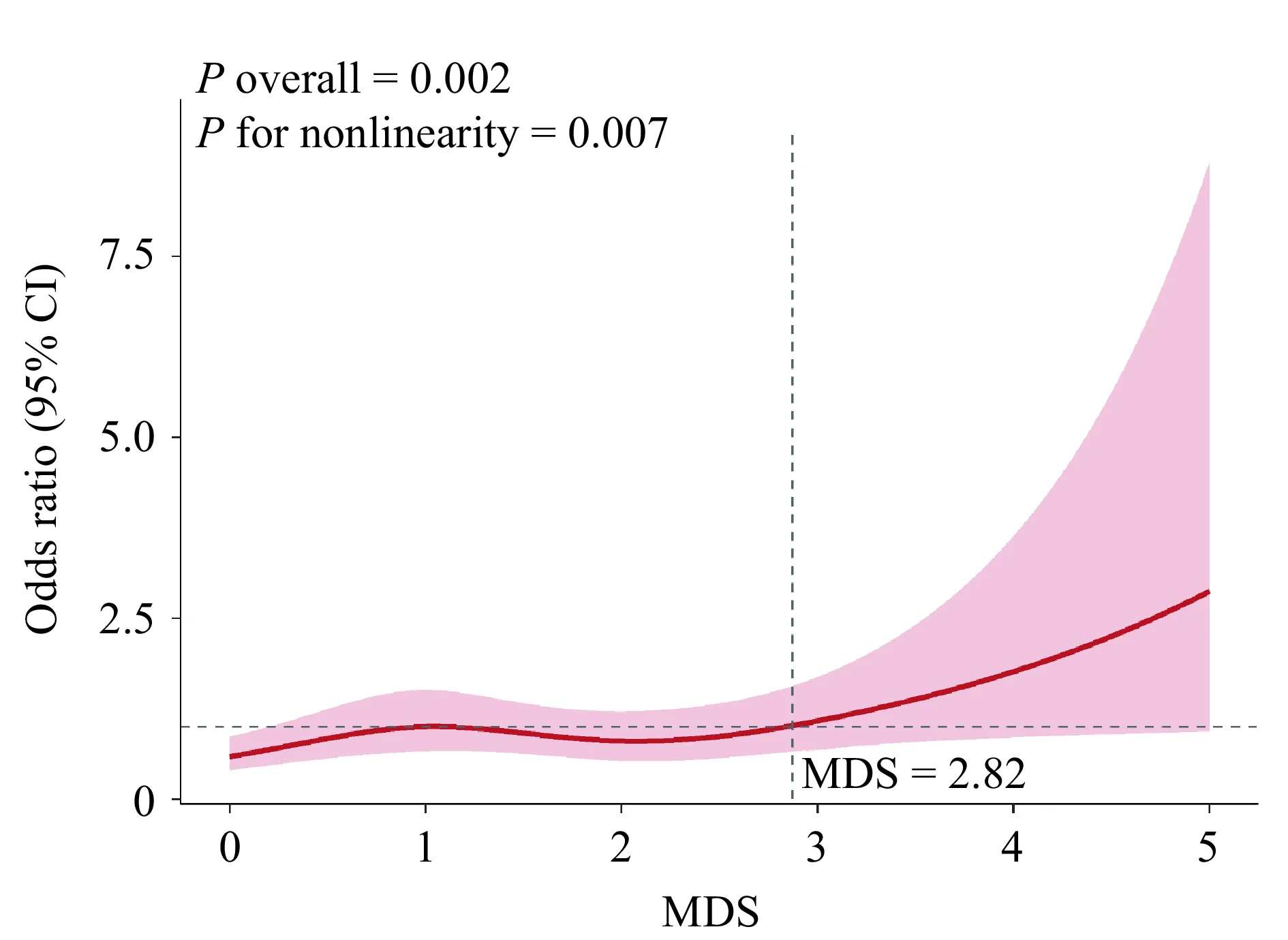
Nonlinear association between MDS and odds of psoriasis. The figure presents the results of an RCS analysis investigating the relationship between MDS and the odds of psoriasis. Abbreviations: MDS, magnesium depletion score; RCS, restricted cubic spline.

**Figure 2:**
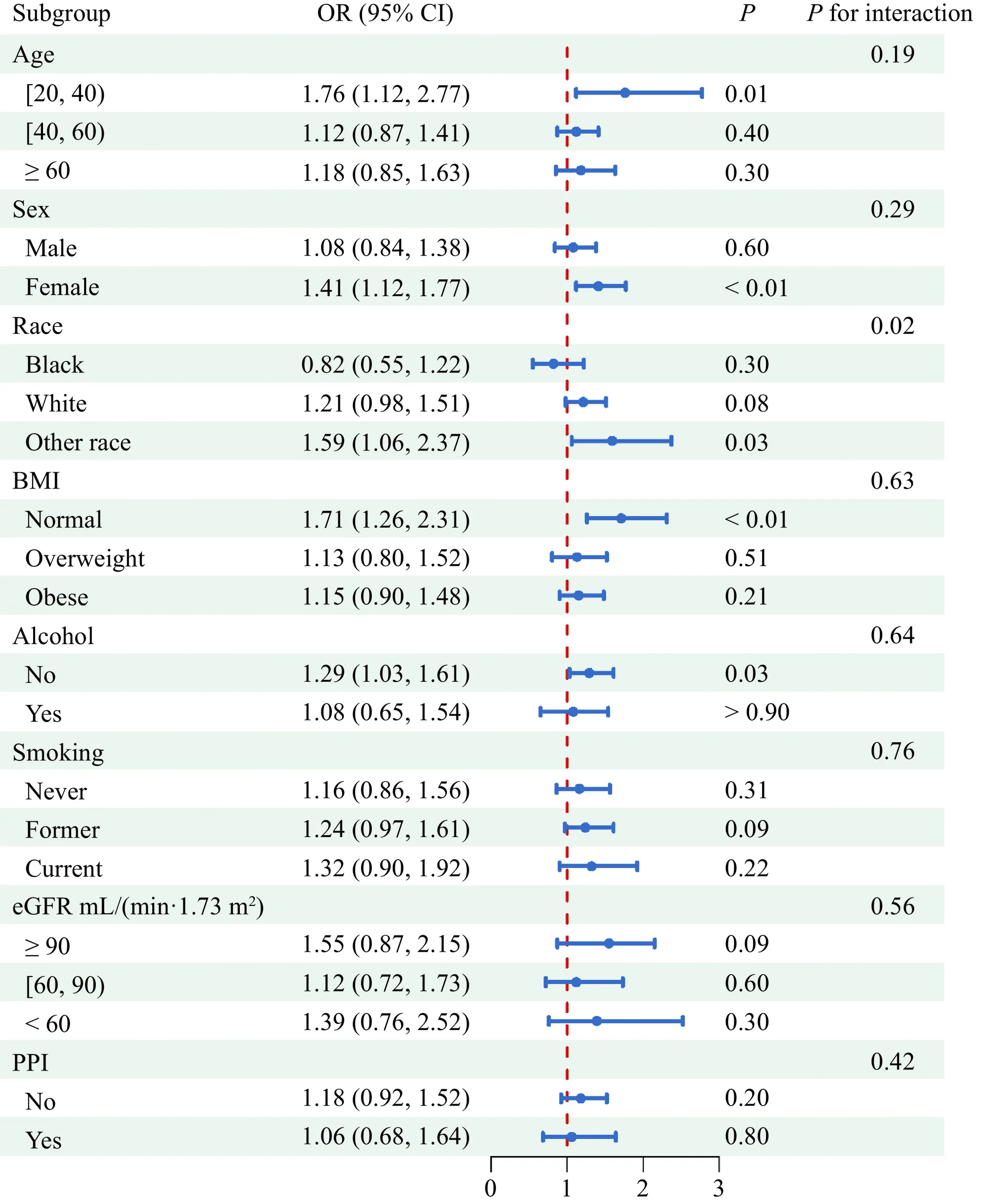
Subgroup analyses and interaction tests of the association between MDS and odds of psoriasis. This chart presents the results of subgroup analysis and interaction test, aiming to explore the influence of different factors on the association between MDS and the odds of psoriasis. Abbreviations: BMI, body mass index; eGFR, estimated glomerular filtration rate; MDS, magnesium depletion score; OR, odds ratio; PPI, proton pump inhibitor.

The current study demonstrates a significant association between a higher magnesium depletion score and greater odds of psoriasis, with the Q4 MDS group associated with nearly twice the odds compared with the Q1 MDS group. However, dietary magnesium intake showed no significant protective effect against psoriasis. These findings underscore the complexity of assessing magnesium status. Our dietary findings do not dispute the recognized importance of magnesium intake; rather, they emphasize the limitations of relying solely on dietary intake as a proxy for physiologically significant systemic magnesium status. Dietary assessments might overlook critical factors that affect bioavailability, absorption, and utilization. Magnesium is crucial for immune regulation; its deficiency promotes a pro-inflammatory state involving elevated IL-6, TNF-α, and NF-κB activation^[7]^—key pathways in psoriasis pathogenesis. Additionally, magnesium deficiency may exacerbate oxidative stress, which mediates inflammatory responses in psoriasis^[8]^. The observed association between MDS and psoriasis suggests that MDS may provide a useful epidemiological indicator of magnesium depletion status in studies of psoriasis, although its potential clinical utility requires prospective validation. Its composite approach, which integrates medication use, renal function, and alcohol consumption, may capture aspects of magnesium depletion not reflected by dietary assessment alone. However, several limitations should be acknowledged. First, the cross-sectional design precludes causal inference and cannot establish the direction of observed associations. Reverse causality also remains possible because psoriasis and its treatment may influence behaviors, medication use, or other factors incorporated into or associated with MDS. Second, dietary magnesium intake was estimated from two 24-hour dietary recalls, which may be subject to recall error and may not fully capture usual long-term intake. Third, unmeasured or residual confounding factors cannot be ruled out. Future prospective and intervention studies are warranted to determine whether magnesium status is prospectively associated with psoriasis development or activity and whether magnesium supplementation provides benefit, particularly among individuals with magnesium deficiency.

Yours sincerely,

Qinyang Cheng^△^, Jiliang Lu^△^, Chaojing Zhou^△^, Chenxingyue Zhang, Ziyue Diao, Zhiqiang Yin^✉^

Department of Dermatology, the First Affiliated Hospital of Nanjing Medical University, 

Nanjing, Jiangsu 210029, 

China. 

^△^These authors contributed equally to this work.

^✉^Corresponding author: Zhiqiang Yin, E-mail: yinzhiqiang@njmu.edu.cn, ORCID, 0000-0002-6510-1051.

## Additional information

The online version contains supplementary material available at http://www.jbr-pub.org.cn/article/doi/10.7555/JBR.39.20250386
