# Supplementary material for: Association of dietary magnesium intake and magnesium depletion score with psoriasis among US adults: A cross-sectional NHANES study
Source: J Biomed Res. 2026 Sep 28;40(5):567–70. doi: 10.7555/JBR.39.20250386 (PMC13620456; doi:10.7555/JBR.39.20250386)
Supplement: Supplementary file 1 [file jbr-40-5-567-S1.pdf]

Available online at www.jbr-pub.org.cn

Open Access at PubMed Central

Journal of Biomedical Research, 2026 40(5): 1–1

Supplementary Data

JBR

Association of dietary magnesium intake and magnesium depletion score with psoriasis among US adults: A cross-sectional NHANES study

Qinyang Cheng△, Jiliang Lu△, Chaojing Zhou△, Chenxingyue Zhang, Ziyue Diao, Zhiqiang Yin✉

Department of Dermatology, the First Affiliated Hospital of Nanjing Medical University, Nanjing, Jiangsu 210029, China.

Supplementary Table 1 **Multivariate logistic regression models of MDS with psoriasis**

Variable	Model 1		Model 2		Model 3	
	OR (95% CI)	*P*	OR (95% CI)	*P*	OR (95% CI)	*P*
MDS	1.30 (1.13, 1.49)	< 0.001	1.25 (1.02, 1.53)	0.033	1.23 (1.02, 1.51)	0.043
MDS category						
Q1	Ref		Ref		Ref	
Q2	1.78 (1.30, 2.44)	< 0.001	1.61 (1.17, 2.21)	0.004	1.62 (1.16, 2.21)	0.006
Q3	1.56 (1.06, 2.40)	0.048	1.38 (0.80, 2.37)	0.310	1.36 (0.78, 2.36)	0.350
Q4	2.35 (1.40, 3.96)	< 0.001	2.10 (1.06, 3.68)	0.027	1.97 (1.14, 3.45)	0.046

Model 1 was adjusted for nothing. Model 2 was adjusted for age, sex, and race. Model 3 was adjusted for age, sex, race, BMI, drinking, smoking status, and diabetes. For MDS category, Q1 indicates MDS = 0, Q2 indicates MDS = 1, Q3 indicates MDS = 2, and Q4 indicates MDS ≥ 3. $P < 0.05$ is considered statistically significant. Abbreviations: BMI, body mass index; CI, confidence interval; MDS, magnesium depletion score; OR, odds ratio; Ref, reference.

△These authors contributed equally to this work.

✉Corresponding author: Zhiqiang Yin, E-mail: yinzhiqiang@njmu.edu.cn, ORCID, 0000-0002-6510-1051.

Received: 07 September 2025; Revised: 16 November 2025; Accepted: 19 November 2025; Available online: 19 November 2025; Published date: 28 September 2026

CLC number: R758.63, Document code: B

The authors reported no conflicts of interest.

This is an open access article under the Creative Commons Attribution (CC BY 4.0) license, which permits others to distribute, remix, adapt and build upon this work, for commercial use, provided the original work is properly cited.

© 2026 by Journal of Biomedical Research.

https://doi.org/10.7555/JBR.39.20250386
